# Intensity-modulated radiation therapy with the central shielding technique for patients with uterine cervical cancer^†^

**DOI:** 10.1093/jrr/rrad039

**Published:** 2023-06-15

**Authors:** Akira Torii, Natsuo Tomita, Mayu Kuno, Masahiro Nishio, Yuki Yamada, Taiki Takaoka, Dai Okazaki, Masanari Niwa, Nozomi Kita, Seiya Takano, Takayuki Murao, Yasutaka Ogawa, Akio Hiwatashi

**Affiliations:** Department of Radiation Oncology, Nagoya City University Hospital, 1 Kawasumi, Mizuho-cho, Mizuho-ku, Nagoya, Aichi 467-8601, Japan; Department of Radiation Oncology, Nagoya City University Hospital, 1 Kawasumi, Mizuho-cho, Mizuho-ku, Nagoya, Aichi 467-8601, Japan; Department of Radiation Oncology, Ichinomiya Municipal Hospital, 2-2-22 Bunkyo, Ichinomiya, Aichi 491-8558, Japan; Department of Radiation Oncology, Kasugai Municipal Hospital, 1-1-1 Takaki-cho, Kasugai, Aichi 486-8510, Japan; Department of Radiation Oncology, Konan Kosei Hospital, 137 Ohmatsubara, Takaya-cho, Konan, Aichi 483-8704, Japan; Department of Radiation Oncology, Nagoya City University Hospital, 1 Kawasumi, Mizuho-cho, Mizuho-ku, Nagoya, Aichi 467-8601, Japan; Department of Radiation Oncology, Nagoya City University Hospital, 1 Kawasumi, Mizuho-cho, Mizuho-ku, Nagoya, Aichi 467-8601, Japan; Department of Radiation Oncology, Nagoya City University Hospital, 1 Kawasumi, Mizuho-cho, Mizuho-ku, Nagoya, Aichi 467-8601, Japan; Department of Radiation Oncology, Nagoya City University Hospital, 1 Kawasumi, Mizuho-cho, Mizuho-ku, Nagoya, Aichi 467-8601, Japan; Department of Radiation Oncology, Nagoya City University Hospital, 1 Kawasumi, Mizuho-cho, Mizuho-ku, Nagoya, Aichi 467-8601, Japan; Department of Radiation Oncology, Ichinomiya Municipal Hospital, 2-2-22 Bunkyo, Ichinomiya, Aichi 491-8558, Japan; Department of Radiation Oncology, Kasugai Municipal Hospital, 1-1-1 Takaki-cho, Kasugai, Aichi 486-8510, Japan; Department of Radiation Oncology, Nagoya City University Hospital, 1 Kawasumi, Mizuho-cho, Mizuho-ku, Nagoya, Aichi 467-8601, Japan

**Keywords:** uterine cervical cancer, intensity-modulated radiotherapy, helical tomotherapy, adverse effects, treatment outcome

## Abstract

We aimed to examine outcomes and toxicities of intensity-modulated radiation therapy (IMRT) with the central shielding (CS) technique for patients with uterine cervical cancer. This retrospective study included 54 patients with International Federation of Gynecology and Obstetrics IB-IVA cancer. Whole pelvic radiotherapy or extended-field radiotherapy were performed at the dose of 50.4 Gy in 28 fractions with helical tomotherapy (HT). Six patients had para-aortic lymph node metastases. The CS technique with HT was utilized after a total dose of 28.8–41.4 Gy to reduce doses to the rectum and bladder. The prescribed dose of intracavitary brachytherapy was mainly 18–24 Gy in three or four fractions at point A. Concurrent chemotherapy was used for 47 patients (87%). Median follow-up time was 56 months. Seventeen patients (31%) developed recurrence. The recurrence of the cervix was observed in two patients (4%). The 5-year rates of the locoregional control, progression-free survival (PFS) and overall survival were 79, 66 and 82%, respectively. Among several factors evaluated, histological type of adenocarcinoma was only a significantly worse prognostic factor for PFS by multivariate analysis (hazard ratio, 4.9 [95% confidence interval, 1.3–18], *P* = 0.018). Grade 2 or higher late toxicities were observed in nine patients (17%). Two patients (4%) each had grade 3 proctitis and grade 3 ileus, respectively. No grade 4 toxicity or treatment-related death was observed. The results suggest that IMRT with the CS technique allows a high local control without increasing the risk of complications for cervical cancer patients.

## INTRODUCTION

Uterine cervical cancer is a leading cause of cancer death in women worldwide [1]. Concurrent chemoradiotherapy is recommended as the primary treatment worldwide for patients with International Federation of Gynecology and Obstetrics (FIGO) IB-IVA cancer when definitive radiotherapy (RT) is selected [2]. In Japan, the central shielding (CS) technique is generally used in whole pelvic RT (WPRT) to reduce the doses to organs at risk (OARs) such as the rectum and bladder [3, 4]. CS technique is usually initiated at the dose of 30–40 Gy of a total dose of 50.4 Gy, and generally depends on the FIGO stage. Japanese studies of conventional RT with the CS technique reported favorable outcomes and acceptable toxicities for patients with cervical cancer [3, 5, 6]. Intensity-modulated radiation therapy (IMRT) improves the dose conformity of the target volume while minimizing the dose delivered to the OARs. Over the past decades, IMRT has improved clinical outcomes for several cancers. Helical tomotherapy (HT) is a novel IMRT technique with image-guided RT (IGRT) [7–9]. We have been using HT for the treatment of cervical cancer. To the best of our knowledge, there are few data currently available on clinical results of definitive IMRT with the CS technique for cervical cancer. Herein, we aimed to examine outcomes and toxicities of IMRT with the CS technique for patients with uterine cervical cancer.

## MATERIALS AND METHODS

### Patient selection and pretreatment assessments

The inclusion criteria of this retrospective study were as follows: (i) histological diagnosis of uterine cervical cancer; (ii) FIGO 2008 stage IB-IVA; (iii) no distant metastasis according to the FIGO 2008 staging system except to the para-aortic lymph node (PALN); (iv) definitive RT at our hospitals between 2012 and 2018; and (v) use of IMRT with the CS technique. Histological type of small cell carcinoma was excluded from this study. Pretreatment assessments included a complete medical history and physical examination, laboratory evaluation, chest X-ray, computed tomography (CT) of the chest, abdomen and pelvis, and magnetic resonance imaging (MRI) of the pelvis. Cystoscopy, recto-sigmoidoscopy and fluorine-18 2-fluoro-2-deoxy-d-glucose-positron emission tomography (PET)-CT scans were optional. Lymph nodes (LNs) with the largest diameter of >10 mm on CT and/or MRI or with significant uptake on PET were defined as metastatic disease. This study was approved by our Institutional Review Board. The final cohort consisted of 54 patients. Table 1 shows patient and treatment characteristics.

**Table 1:** Patient and treatment characteristics

Characteristics	*n* = 54
Age	57 (33–84)
FIGO stage (2008)	
IB1	1 (2%)
IB2	1 (2%)
IIA1	2 (4%)
IIA2	2 (4%)
IIB	33 (61%)
IIIA	1 (2%)
IIIB	13 (24%)
IVA	1 (2%)
Histology	
SCC	49 (91%)
Adenocarcinoma	5 (9%)
Tumor size (cm)	4.4 (2.1–8.5)
Pelvic LN metastases	26 (48%)
Para-aortic LN metastases	6 (11%)
Concurrent chemotherapy	47 (87%)

SCC, squamous cell carcinoma.

Data are shown as *n* (%) or a median (range).

### Radiation therapy

**Fig. 1:**
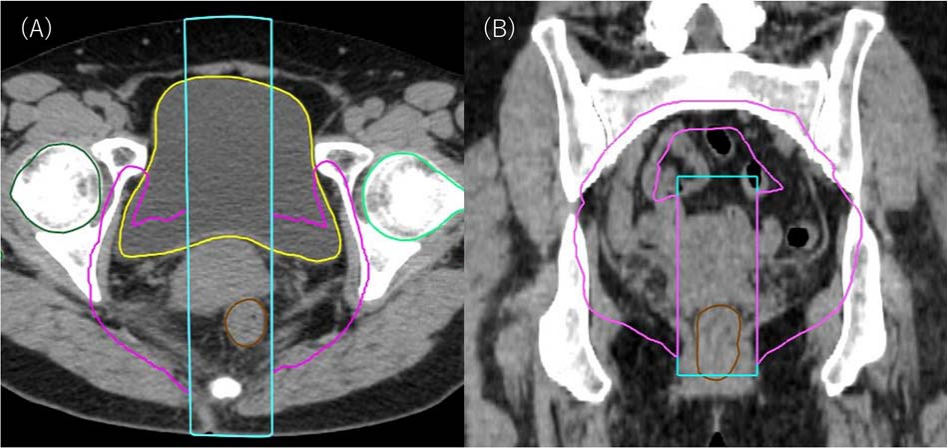
The CS block setting in transverse (**A**) and coronal (**B**) sections. CS block (light blue line) to avoid exposure to the rectum (brown line) and bladder (yellow line) was defined as 3.5–4 cm wide with the same thickness as the body was placed in the center of pelvis. The PTV for CS-WPRT (WPRT) (pink line) was created as PTV for WPRT with the CS block excluded.

**Fig. 2:**
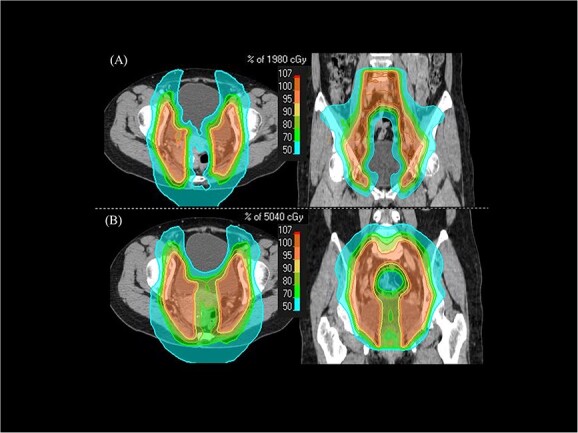
The dose distributions of WPRT with the CS technique (CS-WPRT) (**A**) and WPRT (**B**) consisted of no CS (30.6 Gy) and CS techniques (19.8 Gy) for the patient with FIGO IIB cancer.

All patients were immobilized with custom immobilization devices in a supine position. CT with a 2.5 mm slice thickness was taken. The patient retained urine in their bladder for about 1 h before RT planning CT and each irradiation to reduce the doses to the small bowel. All simulation data were imported into the treatment planning system. Gross tumor volume included any lesion that was visible on a pelvic examination, CT, MRI and PET-CT, if available. Clinical target volume (CTV) was defined and delineated according to the Japanese radiotherapy guidelines for cervical cancer [10, 11]. Primary planning target volume (PTV) covered the primary CTV with a 10–15 mm margin for cephalocaudal and ventrodorsal directions and 7–10-mm margin for lateral directions. A 5-mm isotropic expansion of the prophylactic nodal CTV was added to create the prophylactic nodal PTV. In the patients with pelvic LN and PALN metastases, the involved nodal PTV was created with 10 mm margin around lymph nodal lesion. PTV for WPRT was defined as the combination of the primary PTV, prophylactic nodal PTV and involved nodal PTV. For the treatment planning of CS-WPRT, a 3.5–4.0 cm wide CS block with the same thickness as the body was placed virtually at the center of the pelvis [12]. The cranial side of the CS block was positioned to avoid overlapping with prophylactic PTV. The virtual CS block was removed from the PTV for WPRT (PTV for CS-WPRT) (Fig. 1). OARs included the rectum, bowel, bladder, bilateral femoral head and spinal cord. The kidney, liver and stomach were also included in cases with PALN metastasis. The CT images and structure sets were transferred to the Tomotherapy Hi-Art System workstation (Accuray, Sunnyvale, USA). A superposition algorithm was used for plan calculation. Regarding the tomotherapy treatment conditions, a 2.5 cm field width, pitch of 0.287 and modulation factor of 2.0 were used. The plans were optimized to achieve a target coverage with 50% (D50) of the PTV receiving 100% of the prescription dose, and to provide a dose distribution that was as homogenous as possible within the target. In addition, the plans were optimized using dose-volume histogram (DVH) dose constraints to reduce the dose to OARs to as low as achievable while maintaining PTV coverage. The dose constraints for target and OARs are shown in Table 2. In the optimization for CS-WPRT, the dose of CS block was reduced to meet dose constraints of Table 2 while minimizing the dose to the rectum and bladder as low as possible. Dose constraints for the OARs were evaluated based on the combination with no CS and CS-WPRT. IGRT was necessarily used before every irradiation for setup. Figure 2 shows an example of the dose distributions of CS-WPRT (Fig. 2A) and total WPRT (Fig. 2B) consisted of no CS- (30.6 Gy) and CS- (19.8 Gy) techniques for the patient with FIGO IIB cancer.

**Table 2:** The dose constraint and actual data in WPRT without CS or CS-WPRT

Structure	Parameter ^a^	Dose constraint	Actual data ^b^
PTV for WPRT without CS	1. maximum dose	<107% of prescribed dose	107% (105–111)
	2. D95%	>95% of prescribed dose	95% (90–99)
PTV for CS-WPRT	1. maximum dose	<107% of prescribed dose	108% (105–111)
	2. D95%	>90% of prescribed dose	90% (81–97)
Rectum	1. maximum dose	<51 Gy	49 Gy (41–51)
	2. V40Gy	<40% of the volume	14% (0–43)
Bowel	1. maximum dose	<105% of prescribed dose	105% (103–109)
	2. V35Gy	<40% of the volume	32% (16–41)
Bladder	1. maximum dose	<107% of prescribed dose	103% (94–108)
	2. V45Gy	<50% of the volume	23% (0–48)
Femoral head	V30Gy	<40% of the volume	8% (0–32)
Spinal cord	maximum dose	<45 Gy	37 Gy (27–44)
Pelvic bone	V40Gy	<40% of the volume	36% (29–47)
CS block of CS-WPRT	V50%	<50% of the volume	30% (3–44)

D95%, dose at 95% of the volume; V30Gy, V35Gy, V40Gy, V45Gy, percentage of the volume receiving at least 30 Gy, 35 Gy, 40 Gy and 45 Gy; V50%, the volume receiving 50% of the prescribed dose.

^a^It was assumed that the dose constraints mentioned above were prescribed at 50.4 Gy.

^b^Actual data were described with median and range.

RT was a combination of WPRT and high-dose-rate intracavitary brachytherapy (ICBT). WPRT was performed for all patients, while extended-field RT (EFRT) was employed for six patients (11%) with PALN metastases in pretreatment imaging tests. The total prescription dose for WPRT or EFRT was 50.4 Gy in 28 fractions. In patients with pelvic LN and/or PALN metastasis, 59.36 Gy in 28 fractions was prescribed to the additional PTV using the simultaneous integrated boost technique. One patient received WPRT at a dose of 45 Gy in 25 fractions because of her complication of connective tissue disease. After 28.8–41.4 Gy of WPRT, the CS technique was utilized by the end of RT. After adequate tumor regression by WPRT, ICBT was administered once a week with MultiSource high-dose rate brachytherapy (BEBIG, Berlin, Germany) with a standard applicator set of tandem and ovoids. Three-dimensional image-guided brachytherapy (3D-IGBT) or additional application of interstitial needles was not used for any patients. The ICBT dose was prescribed at 6 Gy to point A with standard loading of the source dwell positions and weighting according to the Manchester System. When the dose of rectal reference point (defined as 5 mm of submucosa) exceeded 5 Gy, a reduction of the point A dose to 5 Gy was allowed. The patients with lower vaginal invasion received ICBT using a vaginal cylinder to deliver a dose of 5 Gy to 5 mm of submucosa in one or two sessions of total ICBT. Equivalent dose in 2 Gy per fraction (EQD2) was calculated in α/β = 10.

### Chemotherapy

Concurrent chemotherapy was used for 47 patients (87%). Chemotherapy was not used for seven patients (13%) who were elderly or refused to receive chemotherapy. Chemotherapy consisted of weekly cisplatin of 40 mg/m^2^ in most patients. Median (range) number of administrations was 5 (3–6) in 41 patients who received weekly cisplatin. Six patients who received 5-FU (700 mg/m^2^ on Days 1–5) and cisplatin (70 mg/m^2^ on Days 5) received two cycles concurrently with RT and three cycles after RT.

### Follow-up and statistical analysis

Patients were followed up every 3 months for the first 2 years and 4–6 months thereafter. A pelvic examination with pap smear was performed each time. Either CT examination of the chest, abdomen and pelvis or pelvic MRI was performed at least every 6 months. The locoregional control (LRC) was defined as the time from the date of definitive RT to local relapse and/or pelvic LN metastasis detected by imaging tests and/or pelvic examinations, and death from any cause. The progression-free survival (PFS) was defined as the time from the date of definitive RT to any clinical relapse and death from any cause. Patients who died prior to clinical relapse were censored in this analysis. Overall survival (OS) was defined as the time from the date of definitive RT to death from any cause. The distributions of LRC, PFS and OS were calculated by the Kaplan–Meier method. The significance of LRC, PFS and OS was examined using the Log-rank test. Cox’s proportional hazards models were used to calculate the hazard ratio (HR) of each factor for PFS in the multivariate analysis. Toxicity was defined in accordance with the National Cancer Institute Common Terminology Criteria for Adverse Events version 4.0. Statistical analyses were performed with EZR, which is a graphical user interface for R (The R Foundation for Statistical Computing) [13]. A *P*-value of <0.05 was considered significant.

## RESULTS

### Data of EBRT and ICBT

Table 2 shows the dose constraints for target and OARs and actual data in external beam radiotherapy (EBRT). The dose constraints for target and OARs were achieved in most patients. The median actual doses at point A and the reference points for rectum and bladder as defined by International Commission on Radiation Units and Measurements (ICRU) Report 38 were 6.0 (range, 5.0–6.0 Gy), 3.4 (range, 1.9–5.1 Gy) and 5.7 Gy (range, 2.6–9.7 Gy), respectively. A combination of doses in WPRT without CS and CS-IMRT and ICBT is shown in Table 3.

**Table 3:** Combination of doses in WPRT and ICBT

	WPRT without	CS-WPRT	ICBT	ICBT	Total EQD2
	CS (Gy)	(Gy)	(Gy)	fractions	(Gy)
IB, IIA	30.6	19.8	24 (12–24)	4 (2 or 4)	62.1 (46.1–62.1)
IIB	30.6 (28.8–41.4)	19.8 (9.0–21.6)	24 (15–24)	4 (3 or 4)	62.1 (53.4–64.7)
IIIA, IIIB, IVA	41.4 (30.6–41.4)	9.0 (9.0–19.8)	22 (14–24)	4 (3 or 4)	63.8 (57.8–70.0)

Data are shown as a median (range).

### Outcomes

**Fig. 3:**
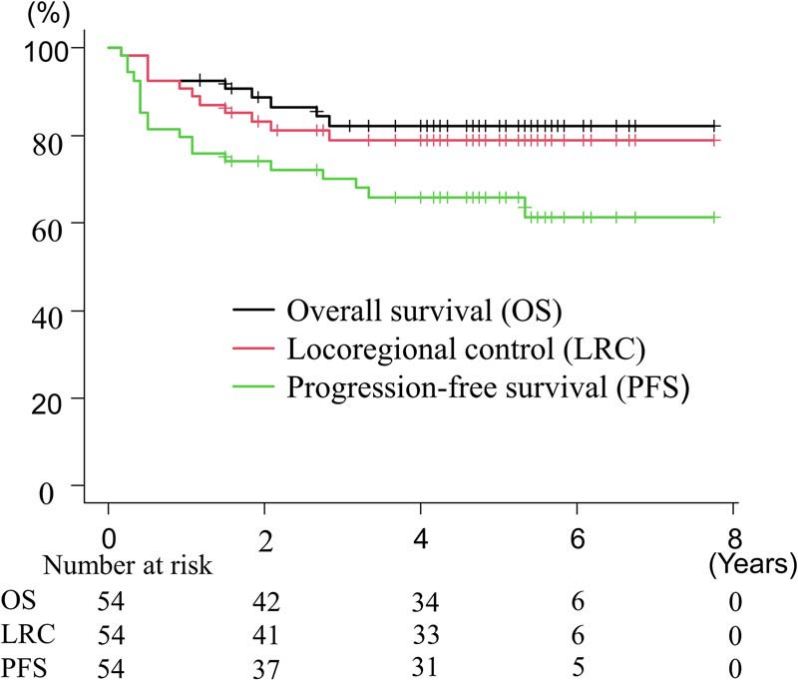
The LRC, PFS and OS.

Median follow-up time was 56 months (range, 2–93). Nine patients (17%) died, consisting of urinary tract infection (*n* = 1), pneumonia (*n* = 1) and cervical cancer (*n* = 7). Seventeen patients (31%) developed recurrence. The primary sites of recurrence were as follows: only cervix (*n* = 1); only pelvic LN (*n* = 1); cervix/ pelvic LN/ PALN simultaneously (*n* = 1); only distant metastases (*n* = 14) (PALN = 1; PALN/lung = 1; supraclavicular LN = 1; inguinal LN = 1; bone = 1; vulva = 1; brain = 1; lung/liver = 2; peritoneum = 5). The 5-year LRC, PFS and OS rates were 79 (95% confidence interval [CI], 65–88%), 66 (95% CI, 51–77%) and 82% (95% CI, 69–90%), respectively. Figure 3 shows survival curves for LRC, PFS and OS.

Table 4 shows the results of the log-rank tests and multivariate analyses for PFS. Patients with FIGO ≥ IIIA and histological type of adenocarcinoma were associated with worse PFS in the log-rank test (*P* = 0.016 and < 0.001, respectively). Among several factors such as FIGO stage, LN metastasis, EQD2 and use of chemotherapy, the results of the multivariate analysis showed that histological type remained a prognostic factor for worse PFS. Adjusted HR of adenocarcinoma for PFS was 4.9 (95% CI, 1.3–18, *P* = 0.018).

**Table 4:** Log-rank test and multivariate analysis evaluating PFS

	Log-rank test	Multivariate analysis	
	*P*-value	HR (95% CI)	*P*-value
Age < 55	0.65	0.75 (0.23–2.4)	0.62
FIGO ≥ IIIA	0.016	1.7 (0.55–5.0)	0.36
Pelvic LN metastasis +	0.073	2.1 (0.60–7.3)	0.25
Para-aortic LN metastasis +	0.44	0.77 (0.19–3.2)	0.72
Adenocarcinoma	< 0.001	4.9 (1.3–18)	0.018
EQD2 < 62	0.72	1.7 (0.42–6.5)	0.47
No use of chemotherapy	0.063	1.9 (0.46–7.5)	0.39

### Toxicity

Late complications are summarized in Table 5. Grade 2 or higher late toxicities were observed in nine patients (17%). Three patients (6%) had a grade 2 pelvic bone insufficiency fracture and two patients (4%) had a grade 2 hematuria. Two patients (4%) had grade 3 proctitis. Two patients (4%) had grade 3 ileus. The rates of grade 2 and grade 3 toxicities were 9 (*n* = 5) and 7% (*n* = 4), respectively. No grade 4 toxicity or treatment-related death was observed.

**Table 5:** Grade 2 or higher late toxicity

	Grade 2	Grade 3	Grade 4	Total
Proctitis	0	2 (4%)	0	2 (4%)
Ileus	0	2 (4%)	0	2 (4%)
Hematuria	2 (4%)	0	0	2 (4%)
Insufficiency fracture	3 (6%)	0	0	3 (6%)
Total	5 (9%)	4 (7%)	0	9 (17%)

## DISCUSSION

In the present study, the 5-year rates of the LRC, PFS and OS were 79, 66 and 82%, respectively. Table 6 shows a summary of clinical outcomes for locally advanced cervical cancer using IMRT or three-dimensional conformal radiotherapy (3DCRT) [6,14–17]. The efficacies of our CS-IMRT method were similar compared with other studies, as shown in Table 6. Especially, the recurrence of the cervix was observed in only two patients (4%) despite about 90% of patients having more than FIGO IIB cancer. Previous studies compared outcomes of definitive IMRT with conventional RT in patients with cervical cancer [18–20]. Most studies showed that OS was not significantly different between IMRT and 3DCRT. This may result from the same prescription dose in these two methods. In IMRT, care should be taken to intrafractional and interfractional organ motion of the uterus, as well as OARs such as the rectum and bladder. Previous studies examined interfractional motion of the uterus during IMRT [21, 22], but they did not establish the proper margin between CTV and PTV. A setup margin of 5 to 10 mm was generally adopted in other studies of definitive IMRT for cervical cancer. We applied a wider margin in view of intrafractional and interfractional motion of the uterus and steep dose distribution with IMRT (10–15 mm margin for cephalocaudal and ventrodorsal directions and 7–10 mm margin for lateral directions between the CTV and PTV). The combination of the wider margin and IGRT may lead to favorable LRC. Furthermore, initial recurrent site of pelvic LN/ PALN was two patients in the present study. Although the benefit of boost irradiation for LN metastasis was unclear, additional administration to LN metastases may improve LRC similarly to other studies [23–25]. Most recurrences (82%) were distant metastases in the present study. One of the reasons may be that PET-CT scan were not routinely performed before treatment. Therefore, potential distant metastasis may be underestimated. Peritoneal dissemination was observed in 5 patients of 14 with distant metastases. This may be caused by about 90% patients having more than FIGO IIB cancer.

**Table 6:** Comparison with clinical outcomes of other reports

Author	*n*	FIGO stage	EBRT technique	Use of CS in EBRT	EBRT dose (Gy)	ICBT technique	ICBT dose	EQD2 (Gy)	OS	PFS	≥G3 late toxicity
Toita *et al*. [6]	60	IB-IIB, 100%	3DCRT	use	50	2D	24Gy/4fx	52	95% at 2 years	90% at 2 years	0%
Chen *et al*. [14]	125	IB-IIB, 61% IIIA-IIIB, 39%	IMRT	non-use	45–54	2D	20–33.5Gy/4-7fx	NA	73.8% at 4 years	67.2% at 4 years	GI, 4% GU, 6%
Mukai *et al*. [15]	30	IB-IIB, 70% IIIA-IVA, 30%	IMRT	use	50.4	2D	25Gy/5fx	71	86.3% at 3 years	83.3% at 3 years	0%
Kusada *et al*. [16]	40	IB-IIB, 90% IIIB-IVA, 10%	3DCRT	non-use	45	3D	18-24Gy/3-4fx	63–69	85% at 2 years	75% at 2 years	GI, 3% GU, 5%
Potter *et al*. [17]	1341	IB-IIB, 75% IIIA-IVB, 25%	3DCRT/ IMRT/	non-use	45–50	3D	NA	90	74% at 5 years	68% at 5 years	GI, 9% GU, 7%
our study	54	IB-IIB, 72% IIIA-IVA, 28%	IMRT	use	50.4	2D	24Gy/4fx	62	82% at 5 years	66% at 5 years	GI 7% GU 0%

NA, not available; GI, gastrointestinal; GU, genitourinary

The incidence of grade 2 late toxicities was low (gastrointestinal [GI], 0%; genitourinary [GU], 4%). This finding may be associated with the excellent dose distribution achieved by IMRT, which may constrain the dose to OARs such as the rectum, bladder and small bowel. Furthermore, accurate setup by IGRT may also have contributed to these favorable results. The CS technique could reduce the doses to OARs and may contribute to the low rates of late toxicities. The rate of grade 3 or higher GU toxicities was 0%, whereas the rate of grade 3 or higher GI toxicities was 7% and was comparable or slightly higher compared with other studies [6,14–17]. One of the causes could be the use of 2D-ICBT. In 2D-ICBT of our current study, the median doses at reference points for the rectum and bladder according to the ICRU Report 38 were 3.4 (range, 1.9–5.1 Gy) and 5.7 Gy (range, 2.6–9.7 Gy), respectively. In JAROG0401/ JROSG04–2, the reported median doses to the rectum and bladder were 4.9 (range, 2.2–10.5 Gy) and 4.8 Gy (range, 2.1–12.1 Gy), respectively. Our ICBT doses, particularly for the rectum, were at the same or slightly lower level compared with those values of JAROG0401/ JROSG04–2. Nevertheless, 7% patients experienced grade 3 or higher GI toxicities. It is also impossible to evaluate doses of the OARs by summing the doses of EBRT and 2D-ICBT. A report comparing IMRT and 3D-IGBT with 2D-EBRT and 2D-ICBT reported that grade 3 or higher late toxicities were significantly lower in the IMRT/3D-IGBT group than 2D-EBRT/ICBT (11 vs 21%, respectively, *P* = 0.02) [26]. In our study, two cases of grade 3 ileus occurred in the sigmoid colon and distal small bowel near the uterus, which may associate with 2D-ICBT that cannot adequately evaluate intestinal dosimetry. Grade 2 fracture occurred in three cases (sacrum = 1, pubis = 1, both sacrum and pubis = 1). All these cases were elderly patients over 70 years old.

There are some clinical studies that compared IMRT and 3DCRT for cervical cancer. A meta-analysis by Lin *et al*. reported that late GU toxicity was significantly lower in IMRT than 3DCRT/ 2DRT (odds ratio = 0.09, 95% CI,0.01–0.67, *P* = 0.02), whereas chronic GI toxicity was not significantly different between the two techniques [27]. Moreover, a previous prospective randomized study by Gandhi *et al*. reported that the IMRT arm had a lower incidence of chronic GI toxicity than a conventional RT arm (13.6 vs 50%, *P* = 0.011) [18].

In Japan, the CS technique is generally used in WPRT to reduce the doses to OARs, such as the rectum and bladder, and to administer the dose to the pelvic LN [3, 4]. However, the clinical use of definitive IMRT in CS-WPRT is uncommon even in Japan. As a planning study, Tamaki *et al*. reported that IMRT could decrease doses to the rectum and bladder and maintain the coverage to the PTV compared with 3DCRT for cervical cancer [28]. In their report, the DVH parameters for WPRT (20 Gy) and CS-WPRT (30 Gy) in IMRT plans were demonstrated. The average V40Gy for the rectum, bowel and bladder was reported to be 6.9, 29.8 and 41.7%, respectively. The prescribed dose component and the use of CS technique were different, but these parameters were at a similar level to our current study (Table 2). In addition, in the prospective report for IMRT without CS, mean V40Gy for the rectum, small bowel and bladder were 42, 31.66 and 42.44%, respectively [18]. Our CS technique seemed to contribute to the reduction of OARs, particularly the rectum. On the other hand, we believe that the greatest merit of using IMRT for WPRT is the reduction of dose to the small bowel. Kidd *et al.* reported a significant reduction in grade 3 or higher bowel or bladder complications with the use of IMRT [20]. However, in Japan, the CS technique with 3DCRT was historically a standard treatment in definitive RT for cervical cancer. The CS technique with 3DCRT was demonstrated in prospective clinical trials (JAROG0401/ JROSG04–2) to achieve excellent local control while reducing rectal doses. Thus, there is no Japanese guideline for IMRT in cervical cancer yet, especially in dose constraints and combination of doses in WPRT and ICBT. Thus, we decided to apply the CS technique based on 3DCRT to IMRT. Mukai *et al*. reported clinical outcomes in a similar RT strategy as the present study [15]. In their study, the 3-year LRC, PFS and OS were 89.9, 83.3 and 86.3%, respectively, with no grade 3 or higher toxicity. The actual doses to the target and OARs were unclear in their study as well as our study because 2D-ICBT was used in both studies. In CS-WPRT with IMRT, doses to the bladder and rectum will decrease, and that of the parametrium increases. Some investigators have tried to develop novel methods of quantitative summation of doses from CS-EBRT with deformable image registration [16, 29, 30].

Some factors limit the interpretation of this single institutional retrospective review. In addition, the sample size was limited to 54 patients. The second limitation associated with this retrospective study was the accuracy of the toxicity assessment. Although the patient charts were thoroughly examined, documents on complications were not described in as much detail as in prospective trials. The third limitation was no dosimetry of entire treatment. It was difficult to evaluate the doses of the target and surrounding OARs because IGBT has not been applied in our institution so far.

In conclusion, the results of the present study suggest that definitive IMRT with the CS technique allows favorable efficacy and acceptable toxicity for patients with locally advanced cervical cancer. In the combination of 2D-ICBT with CS-IMRT, the dose evaluation to the OARs requires attention. A prospective trial is necessary to assess the efficacy and toxicity warrantably in the use of CS-IMRT for locally advanced cervical cancer.

## CONFLICT OF INTEREST

On behalf of all authors, the corresponding author states that there is no conflict of interest.

## FUNDING

This study was supported by a Grant-in-Aid for Research in Nagoya City University Number 2213003.

## DATA AVAILABILITY

The data supporting this study are not publicly available due to the privacy of the research participants, but are available from the corresponding author on reasonable request.

## CLINICAL TRIAL REGISTRATION NUMBER

This study was performed after approval by the institutional review board of Nagoya City University Graduate School of Medical Sciences (approval number: 60-20-0050).
